# Blood donation in times of crisis: Early insight into the impact of COVID‐19 on blood donors and their motivation to donate across European countries

**DOI:** 10.1111/vox.13103

**Published:** 2021-04-09

**Authors:** Torsten Chandler, Sebastian Neumann‐Böhme, Iryna Sabat, Pedro Pita Barros, Werner Brouwer, Job van Exel, Jonas Schreyögg, Aleksandra Torbica, Tom Stargardt

**Affiliations:** ^1^ Hamburg Center for Health Economics University of Hamburg Hamburg Germany; ^2^ Erasmus School of Health Policy & Management Erasmus University Rotterdam Rotterdam the Netherlands; ^3^ Nova School of Business and Economics Lisbon Portugal; ^4^ Erasmus School of Economics Erasmus University Rotterdam Rotterdam the Netherlands; ^5^ Centre for Research on Health and Social Care Management Bocconi University Milan Italy

**Keywords:** blood collection, donor motivation, donor recruitment, donors

## Abstract

**Background:**

In this survey, we aimed to provide early insight into the impact of COVID‐19 on blood donors and their motivation to donate during the crisis.

**Study design and methods:**

We asked representative samples in 7 European countries (Denmark, France, Germany, Italy, Portugal, the Netherlands and the UK) about their blood donation activity and motivation to donate using an online survey. We analysed donor turnout during the COVID‐19 period descriptively and using logistic regression.

**Results:**

Of the 7122 people that responded to the survey, 1205 (16·9%) blood donors were identified, with 33·8% donating during the first 4–5 months of the COVID‐19 period. We observed that around half of donors donated less than normal. The vast majority of donors that did donate made a special effort to do so in response to COVID‐19. The majority of donors were also not aware of their blood being tested for COVID‐19 antibodies. Although the perceived risk of infection among all respondents whilst donating blood was relatively low, those who anticipated a high risk of infection were much less likely to donate (OR = 0·540; *P*‐value = 0·006). Furthermore, those that were adherent to COVID guidelines were also less likely to donate (OR = 0·583; *P*‐value = 0·000).

**Discussion:**

We suggest that blood collection services consider specialist campaigns that focus on the altruistic motivation of donors during the crisis and that they continue to communicate the additional safety measures in place with the aim of reducing the fear of infection whilst donating blood.

## Introduction

The COVID‐19 pandemic has caused an unprecedented impact on blood transfusion and collection with large‐scale disruption to both the supply and demand for blood. Early evidence suggests European countries and across the world have experienced initial drops in whole blood donations, despite centres implementing extra safety measures and public appeals across Europe to encourage continued donation [1, 2, 3, 4, 5, 6, 7]. Research from Hong Kong and China has suggested that anxiety and fear of contracting COVID‐19 were deterrents to donating blood [7, 8], which is consistent with findings from studies on the SARS and avian flu outbreaks [9, 10]. Falling donations have been partly mitigated in the short term by the postponement of elective surgeries, but future demand remains unpredictable and is dependent on how the pandemic evolves [1, 11]. Therefore, maintaining blood supplies remains an important public health concern during the pandemic. In the past, donors have responded well to public appeals to donate in situations of perceived exceptional need, such as the September 11 attacks, mass shootings in the United States and bush fires in Australia where large influxes of donors in short periods of time were documented [11, 12]. Such reactionary spikes have already been observed in some settings across Europe and in Brazil in response to public calls to donate [3, 13, 14, 15]. However, motivations that drive such responses may wane over time and especially so if driven by first‐time donors [12].

Large interruptions to donation activity may have stark consequences for healthcare systems and should be avoided by careful tracking of the supply and demand of blood during these uncertain times. It is therefore essential to gain an initial perspective on the impact of COVID‐19 on blood donors and an understanding of the key aspects of their motivation to donate (or not donate) during this crisis. The aim of this paper was to provide early insight into blood donation activity across seven European countries and the motivation of blood donors to donate or not to donate during the first phase of the COVID‐19 crisis. We do so to understand what is driving changes in donation behaviour and which policies might help to restore donation levels.

## Methods

We asked approximately 7000 people about their blood donation activity and motivation to donate or not to donate within the second wave of the European Covid Survey (ECOS) in June 2020. ECOS is an online survey across seven European countries. Around 1000 people in Denmark, France, Germany, Italy, Portugal, the Netherlands and the UK, representative of their country, participated in the study. The survey was organized in a way to avoid self‐selection, as respondents were not aware of the survey questions beforehand. Participants completed the survey during the period 9–22 June 2020 using online panels provided by the market research company Dynata. Diverse recruiting campaigns reaching out to around 120 000 people were administered online (open recruitment, loyalty programmes, affiliate networks and mobile apps). Survey respondents received payment, which varied depending on the platform they were recruited through. The survey was programmed in the Qualtrics research suite where quotas were implemented to ensure that the country samples matched national census shares, which were representative in terms of age, sex, region and education level. A declaration of compliance for the project was reviewed and approved before the start of the project by the Vice Dean for Research according to the terms of use and ethical standards of the Faculty of Business, Economics and Social Sciences at the University of Hamburg and the European Commission’s RESPECT Code of Practice [16].

### Survey questions and scales

Firstly, we asked all participants whether they had donated blood during the previous 10 years before February 2020 (COVID‐19 period), and for those that answered ‘yes’, we asked how many times they had donated in the 2 years prior to COVID‐19. A timeframe of 10 years was used to capture donors that may have not been active for some time, but could have responded to the crisis by donating blood. Furthermore, we asked whether the entire sample had donated blood since the beginning of February 2020, as this marks the beginning of the COVID‐19 pandemic in Europe. Overall, we were able to identify (a) blood donors, (b) those that donated during COVID‐19 and (c) active donors with at least one donation in the 2 years prior to COVID‐19.

To understand the impact of COVID‐19, we asked donors directly whether they donated less than, more than or the same as they normally would (in the absence of COVID‐19). To gain a better understanding of the motivations underlying the decision to donate, we asked those that stated that they did donate during COVID‐19, about the extent to which they made a special effort to do so in order to help their healthcare system. Possible responses included ‘yes, somewhat’, ‘yes, definitely’, ‘no’ and ‘I don’t know’. The phrasing of the question aimed to identify whether donors saw COVID‐19 as a ‘call to arms’ and responded by helping their healthcare system during the crisis by relieving blood shortages.

As a potential motivator to donate during the pandemic, and following suit from the World Health Organization, we asked all donors how worried they were about their healthcare system being overloaded [17]. Possible responses were on a 5‐point scale from (1) do not worry at all, (2) slightly worry, (3) moderately worry, (4) worry quite a bit and (5) worry a lot. Responses 4 and 5 were combined to create a binary variable for analysis. We included the question as an indicator of how concerned donors were with how their healthcare system was handling the crisis and as a signal for the need for additional support. Despite blood donations not normally being needed directly to treat COVID‐19 patients, blood donation supply concerns during the period were heavily reported in the media [11, 18, 19, 20].

Furthermore, all survey respondents were asked to assess their likelihood of being infected with the novel coronavirus whilst donating blood during the COVID‐19 period (1 = no risk; 5 = very high risk), measuring infection risk as a potential barrier to donation, for example risk of travelling to the donation site, contact with staff and other donors. Lastly, we asked those who donated during the COVID‐19 period if they were aware of being offered and receiving an antibody test, which we considered a potential incentive to donate. Responses included ‘yes, my blood was tested for COVID‐19 antibodies’, ‘no, my blood was not tested for COVID‐19 antibodies’ and ‘I don’t know if my blood was tested for COVID‐19 antibodies’.

### Statistical analysis

The analysis was conducted in two parts (1) a descriptive analysis of blood donors during COVID‐19 and (2) a logistic regression of donation turnout during COVID‐19 among all respondents and active donors.

To analyse the likelihood of donating during the COVID‐19 period, we performed logistic regression analysis across the entire sample of individuals in order to understand which factors were driving the decision to donate during the pandemic. Our dependent variable was binary, indicating whether the sample donated during the COVID‐19 period or not (0 or 1). The focal independent variables of interest were (a) having a perceived high or very high (4 or 5) risk of infection risk whilst donating blood (0 or 1), (b) being worried quite a bit or a lot about the healthcare system being overloaded (0 or 1), (c) whether individuals had a vulnerable person living in their household or not (elderly, disabled or with chronic conditions) (0 or 1) and (d) whether they quite strongly or fully adhered to COVID‐19 guidelines, for example hand washing and social distancing (0 or 1). We expected that individuals with a vulnerable person at home might be dissuaded from donating to avoid exposing them to additional risk, for example through contact with blood donation staff, other donors and those encountered when travelling to the blood donation site. We therefore anticipated that people who were adherent to COVID guidelines would also be less likely to donate blood.

Additionally, we included variables used previously in the literature in our analysis of donation behaviour, which included a measure of donation experience (no. of self‐reported donations in the 2 years prior to the COVID period), age, education level, gender and field of work. We included country fixed effects to account for differences between countries, for example donation eligibility requirements and the structure of country‐specific blood donation systems.

## Results

Of the 7122 people that responded to the survey, 1205 (16·9%) blood donors were identified across the seven European countries. Table 1 presents the characteristics of all survey respondents, and Table 2 presents the sample of blood donors and their background characteristics. Germany, closely followed by France, had the highest number of self‐reported blood donors with 226 (21·5%) and 209 (20·8%) donors, respectively. In contrast, the Netherlands had the lowest number of donors with only 10·2% of the sample.

**Table 1:** Background characteristics of all survey respondents

Country	Overall sample	Number of donors (%)	Age (SE)	Male %	Years in full‐time education (SE)
Germany	1050	226 (21·5%)	48·7 (0·5)	48·6%	12·9 (0·1)
United Kingdom	1041	186 (17·9%)	47·5 (0·5)	46·9%	14·2 (0·1)
Denmark	1005	151 (15·0%)	49·4 (0·5)	48·0%	14·0 (0·1)
The Netherlands	1000	102 (10·2%)	48·1 (0·5)	49·0%	13·0 (0·2)
France	1003	209 (20·8%)	47·7 (0·5)	47·2%	13·8 (0·2)
Portugal	1015	165 (16·3%)	44·6 (0·5)	47·6%	14·2 (0·1)
Italy	1008	166 (16·5%)	48·6 (0·5)	48·0%	14·3 (0·1)
All countries	7122	1205 (16·9%)	47·8 (0·2)	47·9%	13·8 (0·1)

**Table 2:** Background characteristics of blood donors in sample

Country	Number of donors (% of country sample)	**%** donating during COVID‐19 period	Mean age (SE)	% Male	Years in full‐time education. Mean (SE)	No. of donations 2 years pre‐COVID‐19. Mean (SE)	Perceived COVID‐19 infection risk whilst donating blood (SE)*
Germany	226 (21·5%)	35·8%	41·4 (1·0)	54·0%	13·0 (0·4)	4·3 (0·2)	2·28 (0·1)
United Kingdom	186 (17·9%)	40·9%	40·0 (1·1)	45·7%	15·1 (0·4)	4·1 (0·2)	2·44 (0·1)
Denmark	151 (15·0%)	29·8%	43·8 (1·3)	55·0%	14·7 (0·3)	3·8 (0·3)	1·95 (0·1)
The Netherlands	102 (10·2%)	37·3%	42·6 (1·6)	58·8%	12·9 (0·6)	4·5 (0·4)	2·41 (0·1)
France	209 (20·8%)	31·1%	41·3 (1·0)	51·7%	14·3 (0·4)	3·8 (0·2)	2·16 (0·1)
Portugal	165 (16·3%)	21·2%	40·5 (1·1)	52·7%	14·7 (0·3)	3·1 (0·2)	2·03 (0·1)
Italy	166 (16·5%)	40·4%	43·5 (1·2)	57·2%	15·4 (0·4)	4·3 (0·3)	2·26 (0·1)
All countries	1205 (16·9%)	33·8%	41·7 (0·4)	53·1%	14·3 (0·1)	4·0 (0·1)	2·21 (0·0)

* (1 = no risk; 5 = very high risk).

**Fig. 1:**
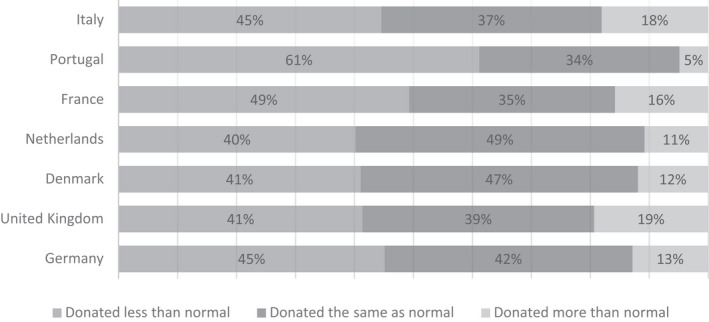
How has COVID‐19 affected your blood donation activity? Self‐reported impact on blood donation activity from 1205 blood donors identified in the sample.

Regarding the impact of COVID‐19 on self‐reported blood donation activity, the results suggest a high number of donors across countries donated less than they normally would compare to their individual reference point, with 61·2% of donors in Portugal selecting this option and around half of donors in France (49·3%) compared with 40‐45% in the remaining countries (Fig. 1). Only a small proportion of donors donated more than they normally would during the COVID‐19 period. Some differences between countries were observed, with only 4·8% of donors in Portugal stating that they donated more than normal, compared with 19·4% in the UK and 18·1% in Italy. In total, however, 407 (33·8%) of the identified donors stated that they donated during the COVID‐19 period of 4–5 months, with some variation between countries, which can be seen in Table 2.

**Fig. 2:**
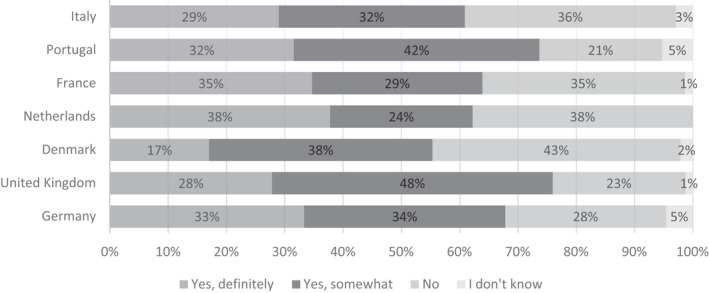
Did you make a special effort to donate in order to help your healthcare system during the COVID‐19 epidemic? Responses from 407 blood donors who donated during COVID‐19.

For donors who donated during COVID‐19 (*n* = 407), we saw that the majority of COVID‐19 donors answered that they ‘yes, somewhat’ or ‘yes, definitely’ made extra effort to donate during the epidemic (Fig. 2). Portugal and the UK reported a high majority, making up 73·7% and 75·9% of donating donors. Furthermore, the Netherlands and France reported a number of donors who responded more explicitly with ‘yes, definitely’, with 37·8% and 34·7% of COVID‐19 donors selecting this option, respectively. Denmark reported a large number (42·6%) of donors who answered ‘no’ to this question.

**Fig. 3:**
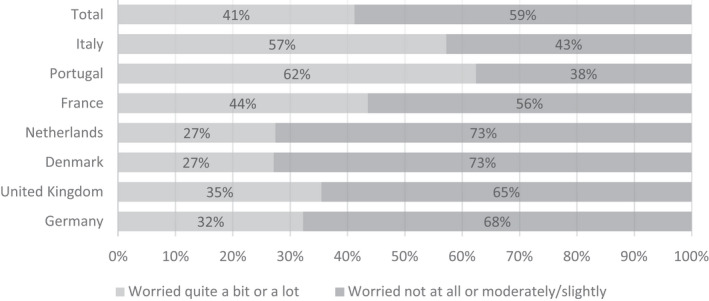
Worries among blood donors about the healthcare system being overloaded. Responses from 1205 blood donors identified in the sample.

In Figure 3, we report how concerned donors were with their healthcare system being overloaded. Those who worried ‘quite a bit’ and ‘a lot’ were compared against the three remaining categories: worrying only moderately, slightly or not at all. We found that although the majority of donors (58·8%) were not worried or only moderately/slightly worried during the period, a substantial group were worried ‘quite a bit’ or ‘a lot’ (41·2%) including the majority of donors in Portugal (62·4%) and Italy (57·2%).

Overall, the mean risk of infection whilst donating blood was perceived to be quite low among donors (M = 2·21 SE = 0·33). We found pairwise differences (one‐way ANOVA with Bonferroni correction) between the UK (M = 2·44, SE = 0·08) and Denmark (M = 1·95, SE = 0·09), *P*‐value = 0·002, and Portugal (M = 2·03, SE = 0·09), *P*‐value = 0·020. Furthermore, we found differences between the Netherlands (M = 2·41, SE = 0·11) and Denmark (M = 1·95, SE = 0·09), *P*‐value = 0·032.

**Fig. 4:**
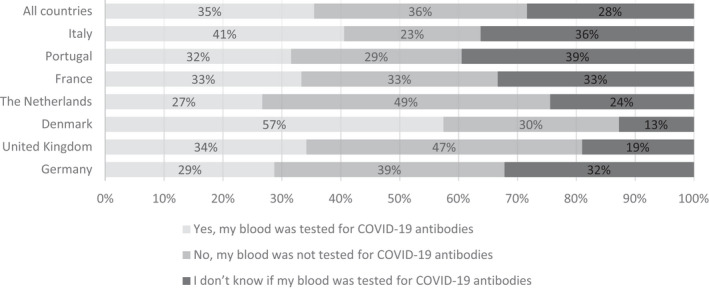
Are you aware whether your blood sample was tested for COVID‐19 antibodies? Responses from 407 blood donors who donated during COVID‐19.

The results suggest that the minority of donors reported to be aware that their blood was tested for COVID‐19 antibodies by their blood collection service (Fig. 4). The exception being the Danish sample where more than half of donors (57.5%) reported being tested. Of those that reported that their blood was tested for COVID‐19 antibodies, 81·3% answered that they had received the results.

The results of the logistic regression analysis of donor turnout during the COVID period are presented in Table 3. Overall, we found that although the perceived risk of infection when donating blood was relatively low, those with a high perceived risk of infection were much less likely to donate (OR = 0·540; *P*‐value = 0·006). Moreover, we identified that those who were quite strongly or fully adherent to COVID guidelines (handwashing, social distancing, etc.) were also less likely to donate (OR = 0·583; *P*‐value = 0·000). We did not find evidence that those who worried about their healthcare system being overloaded were more likely to donate and that having a vulnerable person at home factored in the donation decision. Previous blood donation activity in the 2 years prior to COVID‐19 was a strong predictor of donating during the crisis period, with each additional donation associated with a 87·7% higher donation likelihood (*P*‐value < 0·000). We found that age was important, whereby individuals were incrementally less likely to donate by each age category (compared with 18‐24 years olds). We did not find significant differences between countries in the analysis. Subsequently, we found that those working in the health or education sectors were over twice as likely to donate during COVID‐19 compared with those working in other areas (not including food retail or research).

**Table 3:** Logistic regression analysis of donor turnout during COVID‐19

	Mean (SE)/%	Odds Ratio	SE	z	*P*‐value	95% Confidence Interval	
No. of blood donations two years prior to COVID‐19 period	0·67 (0·02)	1·872	0·043	27·260	0·000	1·790	1·958
Perceived high risk of COVID infection whilst donating blood (4 or 5) (Yes/no)	12·29%	0·540	0·121	−2·750	0·006	0·349	0·838
Worried quite a bit/a lot about healthcare system being overloaded (Yes/no)	41·85%	1·033	0·153	0·220	0·827	0·772	1·382
Vulnerable person in household (Yes/no)	29·30%	0·975	0·161	−0·150	0·877	0·705	1·347
Quite strongly/fully adhered to COVID guidelines (Yes/no)	74·40%	0·583	0·085	−3·690	0·000	0·438	0·777
Age category							
18–24	9·66%	Reference					
25–34	16·22%	0·545	0·109	−3·030	0·002	0·368	0·807
35–44	18·23%	0·352	0·076	−4·830	0·000	0·230	0·538
45–54	17·99%	0·270	0·065	−5·480	0·000	0·169	0·432
55–64	16·29%	0·167	0·048	−6·280	0·000	0·096	0·292
65+	21·60%	0·104	0·033	−7·070	0·000	0·055	0·195
Education (years)							
<10	16·15%	Reference					
10–12	20·31%	0·689	0·164	−1·560	0·118	0·432	1·099
13–15	26·11%	0·692	0·150	−1·700	0·089	0·453	1·057
16–18	22·37%	0·499	0·113	−3·070	0·002	0·320	0·778
18+	15·06%	0·874	0·199	−0·590	0·552	0·559	1·364
Field of work							
Health‐related sector (medical staff, pharmacist, medical student)	8·57%	2·362	0·479	4·240	0·000	1·587	3·514
Education (e.g. schools, nurseries)	7·82%	2·182	0·461	3·690	0·000	1·443	3·301
Food retail (Supermarkets)	3·97%	1·359	0·406	1·030	0·304	0·757	2·441
Research	2·37%	0·913	0·354	−0·230	0·815	0·427	1·954
Other	77·26%	Reference					
Female	52·12%	0·835	0·119	−1·260	0·207	0·632	1·104
Country							
Germany	14·75%	Reference					
United Kingdom	14·62%	1·052	0·249	0·210	0·831	0·662	1·672
Denmark	14·09%	0·647	0·169	−1·670	0·096	0·388	1·079
The Netherlands	14·04%	0·609	0·166	−1·820	0·068	0·357	1·038
France	14·09%	0·845	0·201	−0·710	0·480	0·530	1·348
Portugal	14·26%	0·725	0·192	−1·220	0·224	0·432	1·217
Italy	14·16%	1·083	0·264	0·330	0·745	0·671	1·745
Intercept		0·106	0·031	−7·770	0·000	0·060	0·187

CI, confidence interval; N, 7120; SE, standard error.

Lastly, for the active donor sample (*n* = 992) presented in the Appendix (Tables A1 and A2), we found comparable results whereby those with more donations in the past two years (OR = 1·370; *P*‐value = 0·000) and highly adherent donors were more likely to donate during COVID‐19 (OR = 0·569; *P*‐value = 0·000). However, we did not find evidence that having a high perceived risk of infection whilst donating blood factored into the donation decision (OR = 0·853; *P*‐value = 0·485).

## Discussion

In the study, we found that around half of all donors reported that they donated less than they normally would, which suggests a concerning drop in blood donations throughout the COVID‐19 period across Europe. However, we also identified a small number of donors who donated more than they normally would, perhaps identifying an opportunity to help in a time of need by responding to public appeals.

In terms of donor motivation, we observed that the majority of donors that donated made a special effort to help their healthcare system during the COVID‐19 period. Many of them were also concerned with overcrowding of their healthcare system, indicating that altruistic motives (at least retrospectively) played an important role in donors’ decision to donate and were weighed up against potential risks [5, 21]. Blood donation centres may thus take advantage of this result by appealing to the altruistic nature of donors in times of crisis. As an example, specialist campaigns that focus on the continued need for blood donors during the pandemic could be employed. Such public appeals have been successful in past crises, where large influxes of donors were documented over short periods, for example mass shootings in the United States and bush fires in Australia [11, 12].

Although the perceived risk of infection whilst donating blood was relatively low for many respondents, those who anticipated a high risk of infection were much less likely to donate. Our results coincide with previous research from Hong Kong and China that found anxiety and fear of contracting COVID‐19 were deterrents to donating blood [7, 8], and with studies on the SARS and avian flu outbreaks [9, 10]. Therefore, we suggest that blood donation centres emphasize the steps that have been taken to reduce risk of infection in order to maintain confidence in their services, for example handwashing, face coverings and social distancing. Key messages could assure the public that they can still safely donate blood whilst adhering to COVID‐19 measures. We found that the result did not hold for the active donor sample, which may be explained by a high level of intrinsic donor motivation, even in those that anticipated a high infection risk whilst donating.

Despite all European countries being eventually affected by COVID‐19, there were notable differences in the severity of the crisis and the government response. Italy was hit particularly hard early in the pandemic and was the first European country to apply interventional measures from the beginning of March 2020 in response to the outbreak of the virus [22]. Other EU countries followed soon after by introducing measures from around mid‐March 2020 [22]. Despite this variation, we did not find evidence of country differences in our analysis of donor turnout, which suggests that the results are generalizable across Europe and states may face similar challenges in managing blood supply during the period.

With some countries including Finland, Germany and the United Kingdom discussing the use of ‘immunity passports’ [23, 24, 25], we conjecture that having evidence of COVID‐19 antibodies could be valuable to many individuals and provide reassurance that they are not putting loved ones and those in their community at risk. However, according to our results, the low number of donors who were aware of COVID‐19 antibody tests alongside their donation suggests that the tests were not a relevant incentive for donors. However, by framing such tests as incentives more explicitly, in line with free health checks, which have been used previously to motivate blood donation, donors may be more encouraged to donate [26, 27].

As has been observed in a German setting, many donors prefer to visit at lunchtime and after work in normal times [28]. We speculate that, in addition to perceived infection risk, other factors contributed to a drop in donations that warrant further research. For instance, lockdown and quarantine periods preventing normal movement, changing working conditions disrupting established routines, uncertainty around whether blood collection services were open and the cancellation of blood drives. Further research would allow blood donation centres to accommodate new donation patterns as they begin to observe them. For example, some donors may benefit from extended opening hours and flexibility as previous childcare arrangements (e.g. nurseries and schools) have been disrupted, whilst others may benefit from more blood drive locations in city suburbs to accommodate working from home.

The study findings should be considered in the light of some limitations: firstly, we did not know the blood type of the survey respondents and could therefore not incorporate it into our analysis. However, as our sample is representative across major characteristics, we do not expect large deviations in the ABO or Rhesus factor blood type from the respondents. Secondly, our data are self‐reported, which may explain our donation rate of 5·7%, which was slightly higher than has been reported previously across Europe with figures ranging between 2·5% and 5·4% [29, 30, 31, 32, 33]. In line with other studies, we found our donors to be mostly male (53·1%) and between 30 and 44 (35·3%). We identified a higher proportion of younger donors than was reported in a large European survey, which is likely due to differences in donor definitions, for example donated in lifetime versus in the last 10 years [34].

In conclusion, we observed that despite half of donors donating less than they normally would during the pandemic, most that did donate made a special effort to do so. Furthermore, survey respondents who anticipated a high risk of infection were much less likely to donate. This change in donation behaviour and the associated motivations of donors are relevant to policymakers who are concerned with maintaining adequate blood supply during this crisis. We suggest that blood collection services consider specialist campaigns that focus on the altruistic motivation of donors during the crisis, and they continue to reassure donors of the safety measures in place in their centres. Lastly, the majority of donors appear to have not been incentivized by COVID‐19 antibody tests, which should be considered if framed alongside free health checks as an incentive to elicit blood donations.

## Conflict of interests

The authors declare no conflict of interests.

## Author contributions

TC analysed the data and wrote the original manuscript. SNB, IS, PPB, WB, JVE, JS, AT and TS collaborated in developing the survey questions and provided substantial comments that were incorporated into the manuscript.

## Funding

This work was supported by the European Union's EU Framework Programme for Research and Innovation Horizon 2020 under Grant Agreement No. 721402.

## Appendix

**Table A1:** Background characteristics of active donors*

Country	Number of active donors (% of country sample)	% donating during COVID‐19 period	Mean age (SE)	% Male	Years in full‐time education. Mean (SE)	No. of donations 2 years pre‐COVID‐19. Mean (SE)	Perceived COVID‐19 infection risk whilst donating blood (SE)^**^
Germany	192 (18·3%)	41·7%	39·4 (1·1)	54·2%	12·8 (0·4)	5·0 (0·2)	2·4 (0·1)
United Kingdom	154 (14·8%)	48·1%	38·6 (1·2)	48·1%	14·9 (0·4)	4·9 (0·2)	2·5 (0·1)
Denmark	114 (11·3%)	38·6%	40·2 (1·4)	53·5%	15·0 (0·4)	5·0 (0·3)	2·1 (0·1)
The Netherlands	79 (7·9%)	48·1%	39·0 (1·7)	63·3%	13·0 (0·7)	5·8 (0·4)	2·6 (0·1)
France	178 (17·7%)	36·0%	39·9 (1·1)	51·1%	14·2 (0·4)	4·5 (0·2)	2·2 (0·1)
Portugal	131 (12·9%)	24·4%	38·8 (1·2)	50·4%	15·0 (0·3)	3·9 (0·2)	2·0 (0·1)
Italy	144 (14·3%)	46·5%	41·3 (1·2)	56·3%	15·8 (0·4)	4·9 (0·3)	2·0 (0·1)
All countries	992 (13·9%)	40·2%	39·6 (0·5)	53·1%	14·4 (0·2)	4·8 (0·1)	2·3 (0·0)

* At least one donation in two years pre‐COVID‐19 period.

** 1 = no risk; 5 = very high risk).

**Table A2:** Logistic regression analysis of active donor turnout during COVID‐19

	Mean (SE)/%	Odds Ratio	SE	z	*P*‐value	95% Confidence Interval	
No. of blood donations two years prior to COVID‐19 period	4·80 (0·096)	1·370	0·040	10·730	0·000	1·294	1·451
Perceived high risk of COVID infection whilst donating blood (4 or 5) (Yes/no)	16·13%	0·853	0·194	−0·700	0·485	0·546	1·333
Worried quite a bit/a lot about healthcare system being overloaded (Yes/No)	42·54%	1·100	0·177	0·590	0·555	0·802	1·507
Vulnerable person in household (Yes/no)	23·49%	0·964	0·177	−0·200	0·842	0·673	1·381
Quite strongly/fully adhered to COVID guidelines (Yes/no)	63·81%	0·569	0·092	−3·500	0·000	0·415	0·781
Age category							
18–24	15·32%	Reference					
25–34	28·83%	0·511	0·119	−2·880	0·004	0·323	0·807
35–44	21·77%	0·416	0·103	−3·540	0·000	0·256	0·676
45–54	16·33%	0·366	0·100	−3·670	0·000	0·214	0·626
55–64	10·18%	0·284	0·090	−3·990	0·000	0·153	0·527
65+	7·56%	0·202	0·074	−4·390	0·000	0·099	0·412
Education (years)							
<10	14·82%	Reference					
10–12	16·43%	0·795	0·218	−0·840	0·403	0·465	1·360
13–15	24·19%	0·945	0·236	−0·230	0·821	0·579	1·543
16–18	24·09%	0·616	0·158	−1·900	0·058	0·373	1·017
18+	20·46%	1·033	0·269	0·120	0·902	0·620	1·719
Field of work							
Health‐related sector (medical staff, pharmacist, medical student)	13·41%	1·866	0·433	2·690	0·007	1·184	2·941
Education (e.g. schools, nurseries)	10·79%	1·751	0·428	2·290	0·022	1·085	2·826
Food retail (Supermarkets)	4·94%	0·997	0·343	−0·010	0·992	0·508	1·956
Research	4·33%	0·772	0·296	−0·670	0·500	0·364	1·637
Other	66·53%	Reference					
Female	46·88%	1·077	0·168	0·470	0·636	0·793	1·462
Country							
Germany	19·35%	Reference					
United Kingdom	15·52%	1·154	0·296	0·560	0·575	0·698	1·909
Denmark	11·49%	0·770	0·217	−0·930	0·354	0·443	1·338
The Netherlands	7·96%	0·879	0·276	−0·410	0·680	0·475	1·624
France	17·94%	0·806	0·201	−0·870	0·386	0·495	1·313
Portugal	13·21%	0·663	0·190	−1·430	0·152	0·378	1·163
Italy	14·52%	1·382	0·362	1·230	0·217	0·827	2·309
Intercept		0·458	0·160	−2·240	0·025	0·231	0·908

CI, confidence interval; N, 992; SE, standard error.
